# 中医药调控肿瘤相关巨噬细胞防治肺癌转移的基础研究进展

**DOI:** 10.3779/j.issn.1009-3419.2024.106.14

**Published:** 2024-07-20

**Authors:** Shihui LIU, Jiaxuan LI, Zujun QUE, Pan YU, Jianhui TIAN

**Affiliations:** ^1^200071 上海，上海中医药大学附属市中医医院肿瘤临床医学中心; ^1^Clinical Oncology Center, Shanghai Municipal Hospital of Traditional Chinese Medicine, Shanghai University of Traditional Chinese Medicine, Shanghai 200071, China; ^2^200071 上海，上海中医药大学附属市中医医院肿瘤研究所; ^2^Institute of Oncology, Shanghai Municipal Hospital of Traditional Chinese Medicine, Shanghai University of Traditional Chinese Medicine, Shanghai 200071, China

**Keywords:** 肿瘤相关巨噬细胞, 极化, 肺肿瘤, 转移, 中医药, Tumor-associated macrophages, Polarization, Lung neoplasms, Metastasis, Traditional Chinese medicine

## Abstract

肺癌是全球死亡率最高的癌种，而转移则是导致肺癌患者死亡的主要原因，且防控效率不高。近年来研究发现，免疫疗法或许是突破转移瓶颈的方向。巨噬细胞作为固有免疫的重要组成部分，参与肿瘤发生发展的全过程。肿瘤相关巨噬细胞（tumor-associated macrophages, TAMs）是肿瘤微环境（tumor microenvironment, TME）中最丰富的免疫群体，具有抗肿瘤的M1型和促肿瘤的M2型，后者通过促进肿瘤侵袭和转移、血管和淋巴管生成、免疫抑制和介导播散肿瘤细胞（disseminated tumor cells, DTCs）休眠重激活等途径促进肿瘤转移。近年来，中医药抑制肿瘤转移疗效显著并得到诸多验证，它能通过减少TAMs的募集、抑制M2型极化和调节TME中的细胞因子和蛋白发挥抗肿瘤作用。本文综述了TAMs与肺癌转移之间的关系，梳理了中医药调控TAMs防治肺癌转移的靶点与机制，以期为肺癌防治提供思路。

## Abstract (en)

Lung cancer is the leading cause of cancer-related deaths worldwide, with metastasis being the primary cause of mortality in lung cancer patients, and its prevention and control efficacy remain limited. In recent years, immunotherapy has emerged as a promising direction for overcoming the bottleneck of metastasis. Macrophages, as essential components of innate immunity, participate in the entire process of tumor initiation and progression. Tumor-associated macrophages (TAMs) represent the most abundant immune population in the tumor microenvironment (TME), displaying both anti-tumor M1-like and pro-tumor M2-like phenotypes. The latter promotes tumor invasion and metastasis, angiogenesis, lymphangiogenesis, immune suppression, and reactivation of dormant disseminated tumor cells (DTCs), thereby facilitating tumor metastasis. In recent years, traditional Chinese medicine (TCM) has shown significant efficacy in inhibiting tumor metastasis and has been extensively validated. It exerts anti-tumor effects by reducing the recruitment of TAMs, inhibiting M2-like polarization, and modulating cytokines and proteins in the TME. This paper reviews the relationship between TAMs and lung cancer metastasis, elucidates the targets and mechanisms of TCM in regulating TAMs to prevent and treat lung cancer metastasis, aiming to provide insights into lung cancer prevention and treatment.

最新流行病学数据显示，肺癌成为恶性肿瘤中发病率和死亡率均位居首位的癌种，占恶性肿瘤死亡总数的28.5%^[1]^，而超过90%的死亡是由转移引起^[2]^。如何有效控制肺癌转移，是临床中亟待解决的难题。免疫检查点抑制剂的使用让肺癌晚期患者生存获益，使得免疫疗法成为当前的研究热点。然而，目前肺癌免疫治疗主要针对适应性免疫的T淋巴细胞，由于靶点蛋白表达的限制、自发性和获得性耐药以及不良反应等因素，导致其临床响应率较低^[3]^，并且针对先天性免疫检查点的相关研究仍无突破性进展，促使研究者们把目光逐渐转向固有免疫^[4]^。巨噬细胞是固有免疫的重要组成部分，广泛分布于人体各组织当中，具有丰富的表型和高度的可塑性，参与肿瘤发生发展的全过程。从巨噬细胞招募、增殖、极化、血管生成到免疫抑制，均可作为治疗肺癌转移的潜在靶点^[5]^。因此，靶向巨噬细胞防治肺癌转移，具有较大的研究前景与价值。

中医药疗法来源于中华民族几千年人用经验的积累，是除放化疗外的一种辅助治疗方式，在肿瘤的治疗过程中可以起到协同抑瘤、优势互补、减毒增效的作用，许多抗肿瘤的天然药物也来源于中药^[6]^。随着现代医学的发展，中医药在肿瘤治疗领域的作用得到越来越多的证实和认可。中医药可以通过多靶点调控免疫功能，激活免疫细胞，增强抗肿瘤药物的疗效，并且减少放化疗造成的不良反应。除了直接对肿瘤细胞发挥细胞毒性作用，中药及其有效成分还在肿瘤微环境（tumor microenvironment, TME）中发挥各种免疫调节作用，包括减少肿瘤相关巨噬细胞（tumor-associated macrophages, TAMs）的招募、抑制TAMs向M2型极化、调节TME中的细胞因子和蛋白等^[7]^。因此，本综述聚焦于TAMs与肺癌转移之间的关系，梳理国内外研究进展，探讨中医药调控TAMs防治肺癌转移的相关靶点与机制，以期为肺癌防治提供思路。

## 1 TAMs简介

巨噬细胞包括组织驻留巨噬细胞（tissue-resident macrophages, TRMs）和来源于骨髓造血干细胞的单核巨噬细胞。巨噬细胞可极化为两种状态，一种是经典激活的M1型巨噬细胞，另一种是替代激活的M2型巨噬细胞。M1型巨噬细胞主要通过分泌白细胞介素6（interleukin-6, IL-6）、肿瘤坏死因子α（tumor necrosis factor-α, TNF-α）和诱导型一氧化氮合酶（inducible nitric oxide synthase, iNOS）等促炎性细胞因子，以及产生一氧化氮（nitric oxide, NO）和活性氧（reactive oxygen species, ROS）^[8]^，参与促炎症反应；并且可通过吞噬作用和分泌细胞因子，直接或间接地杀伤肿瘤细胞。M2型巨噬细胞则通过分泌IL-10、转化生长因子-β（transforming growth factor-β, TGF-β）和TNF-α等细胞因子，促进肿瘤血管生成，推动肿瘤的发生发展。

TAMs是浸润于肿瘤组织中的巨噬细胞，由肿瘤所在组织自身存在的TRMs和从外周血招募到肿瘤组织中的骨髓衍生单核细胞组成^[9]^。TAMs是TME中的重要组成部分，也是TME中最丰富的免疫群体，具有从抗肿瘤到促肿瘤的异质性^[10]^。当它被适当激活时，抗肿瘤的M1型TAMs保留了抗原呈递细胞的特性，包括高表达主要组织相容性复合体II类（major histocompatibility complex II, MHC II），分泌促炎细胞因子，促进体内非己成分的清除，支持和激活适应性免疫细胞等^[11]^，对预防肿瘤的发生起重要作用。相反，促肿瘤的M2型TAMs具有免疫抑制作用，低表达MHC II，表达抑制分子如程序性死亡受体1（programmed cell death 1, PD-1）、免疫抑制程序性细胞死亡配体1（programmed cell death ligand 1, PD-L1）、T细胞活化V域免疫球蛋白抑制因子（V-domain immunoglobulin suppressor of T cell activation, VISTA）、免疫调节蛋白B7-H4和T细胞免疫球蛋白黏蛋白-3（T cell immunoglobulin and mucin domain 3, TIM-3）^[12⇓⇓⇓⇓-17]^，在血管生成、细胞外基质（extracellular matrix, ECM）重塑、肿瘤细胞增殖、转移和免疫抑制，以及对化疗和免疫治疗的耐药性等方面发挥着重要作用^[18]^。TAMs的双向调节作用使它逐渐被研究者们所关注。

## 2 TAMs极化的机制

TAMs群体处于M1型和M2型两种状态之间不断过渡的状态，而极化为不同表型是由TME中不同的刺激信号所决定的^[19]^。在肺癌早期，基质细胞分泌集落刺激因子1（colony stimulating factor 1, CSF-1）募集巨噬细胞，TME中主要以发挥抗肿瘤作用的M1型巨噬细胞为主；在肺癌晚期，肿瘤细胞分泌CC趋化因子配体2（chemokine C-C motif ligand 2, CCL2）和表皮生长因子（epidermal growth factor, EGF）诱导巨噬细胞向M2型极化，发挥促肿瘤作用^[20]^。TME中产生的细胞因子和趋化因子还包括IL-1β、CXCL12、血管内皮生长因子（vascular endothelial growth factor, VEGF）等，通过受体介导途径激活整合素α4β1，促进骨髓细胞募集并转化为TAMs^[21]^。缺氧状态下，肿瘤细胞产生的抑瘤素、IL-6、TGF-β等细胞因子可将巨噬细胞极化为M2型^[22]^。Chen等^[23]^还发现肺腺癌细胞分泌的外泌体可将miR-19b-3p递送至TAMs中，靶向抑制蛋白酪氨酸磷酸酶受体PTPRD介导的信号转导因子和转录活化因子3（signal transducer and activator of transcription 3, STAT3）去磷酸化，导致STAT3激活，诱导巨噬细胞向M2型极化。Zhou等^[24]^发现富含miR-184-3p的肿瘤细胞来源的外泌体可被巨噬细胞吞噬，通过靶向早期生长应答因子（early growth response 1, EGR1）抑制c-Jun氨基末端激酶（c-Jun N-terminal kinase, JNK）信号通路，从而诱导巨噬细胞向M2型极化，协同促进肿瘤进展。

## 3 TAMs促进肿瘤转移的机制

现有研究^[25]^结果表明，TAMs能够促进肿瘤细胞增殖、刺激肿瘤血管生成、诱导肿瘤免疫耐受、增强肿瘤细胞的侵袭及转移能力等。因此，深入研究和理解TAMs在肿瘤发生发展中的作用机制，对于制定更有效的肿瘤治疗策略具有重要意义。

### 3.1 TAMs促进肿瘤侵袭和转移

TAMs的促肿瘤功能是多种多样的，并在肿瘤发展的不同阶段中都起作用。在起始阶段，TAMs释放NO和活性氧中间体（reactive oxygen intermediates, ROI），引起DNA损伤和遗传不稳定。TAMs产生EGF和多种介质，如IL-6、人类生长因子（human growth factor, HGF）和糖蛋白非转移性黑色素瘤蛋白B（glycoprotein nonmetastatic melanoma protein B, GPNMB），支持肿瘤干细胞扩增；在后期，TAMs通过释放IL-1和TGF-β促进转移扩散^[26]^。在肿瘤基质中，TAMs产生基质金属蛋白酶（matrix metalloproteinases, MMPs）和尿激酶纤溶酶，促进ECM的降解和重塑，并通过分解黏附分子诱导上皮间充质转化（epithelial-mesenchymal transition, EMT），削弱肿瘤细胞的紧密连接^[27]^，从而促进肿瘤细胞的侵袭和转移。肺癌细胞发生EMT后，将会分泌更多的CCL2以招募并极化TAMs，使其浸润到TME中^[28]^。

### 3.2 TAMs促进血管和淋巴管生成

肿瘤细胞的主要特征之一是诱导血管和淋巴管生成，这也是肿瘤向远处转移扩散的标志。TAMs通过两种方法调节肿瘤血管和淋巴管生成，分别是旁分泌模式和细胞自主模式^[29]^。随着肿瘤的增殖，TME中氧气供应不足，巨噬细胞被募集到肿瘤和间质细胞之间血管化较差的区域，在缺氧诱导因子的刺激下，TAMs释放一组血管生成因子，如VEGF-α、TGF-β、CXCL12、血小板源性生长因子（platelet-derived growth factor, PDGF）和MMPs，促进肿瘤血管生成^[30]^。同时，巨噬细胞在缺氧条件下传递更多的血管内皮生长因子受体（vascular endothelial growth factor receptor, VEGFR），与TME中的VEGF结合，从而影响下游通路并促进TAMs向M2型转化^[31]^。此外，在炎症因子的刺激下，巨噬细胞来源的淋巴管内皮细胞祖细胞可以分化为淋巴内皮细胞，促进已有的淋巴管生长和新的淋巴细胞生成^[32]^。

### 3.3 TAMs促进免疫抑制

TAMs还是TME中免疫抑制的驱动因素。研究^[33]^发现，TAMs通过释放TGF-β、IL-10和精氨酸酶1（arginase-1, Arg-1）等免疫抑制分子，抑制T细胞特异性反应。TAMs可以分泌IL-10诱导T细胞表达抑制性受体，如PD-1和细胞毒性T淋巴细胞抗原4（cytotoxic T lymphocyte-associated antigen-4, CTLA4），这些表面受体与其相应配体PD-L1和CD80/CD86结合导致T细胞免疫应答的负调控，包括细胞凋亡和衰竭^[34]^。TAMs还可以产生CCL17、CCL22和CCL24等细胞因子，抑制CD4^+ ^T和CD8^+ ^T细胞效应器功能，并将调节性T细胞（regulatory T cells, Tregs）募集到TME^[35⇓-37]^，降低免疫应答。

### 3.4 TAMs介导DTCs休眠重激活

靶器官中播散肿瘤细胞（disseminated tumor cells, DTCs）休眠重激活是转移发生的决定性环节。靶器官中的DTCs由于受到微环境、免疫监视、缺氧、血管生成等因素的影响而进入休眠状态^[38]^，潜伏在患者体内，只有当休眠的DTCs被激活后增殖形成肿瘤病灶并被影像学手段检出时，患者方可被诊断为临床转移。其中DTCs的休眠主要涉及磷脂酰肌醇3-激酶（phosphoinositide 3-kinase, PI3K）/丝氨酸/苏氨酸激酶（serine/threonine kinase, Akt）、Notch、Wnt、BMP和细胞外调节蛋白激酶/丝裂原活化蛋白激酶p38（extracellular regulated kinase/p38 mitogen-activated kinase, ERK/p38 MAPK）等信号通路的调控^[39]^。M2型巨噬细胞通过分泌细胞因子激活休眠的DTCs增殖，其中IL-6、IL-10、CCL2、CX3CL1、IL-8与肺癌转移密切相关^[40]^。

## 4 中医药调控TAMs抑制肺癌转移

中医认为疾病的发生是正邪相争的过程，以扶正与祛邪辨证论治为治疗原则。在肺癌发生的初期，人体正气尚存，邪气初生，此时宜利用正气祛邪外出，故常使用清热解毒、散结消肿、活血化瘀、利水渗湿之类的中药。研究^[41]^表明，这些具有“祛邪”作用的中药可以抑制巨噬细胞向肿瘤细胞募集或抑制巨噬细胞极化。在肺癌进程的后期，人体正气亏虚，无力抗邪，此时治则应当以扶正为主，多使用补益正气、健脾益气、温阳补肾、养血安神之类的中药。研究^[41]^表明，这些具有“扶正”作用的中药可以通过调节TAMs相关的细胞因子和蛋白，改善免疫抑制微环境。

### 4.1 减少TAMs的募集

肺癌原发灶的肿瘤细胞可以通过分泌外泌体、整合素和趋化因子等，招募外周血中的巨噬细胞浸润至TME成为TAMs^[42]^。参与TAMs募集的分子包括TGF-β、CSF-1、CCL2、IL-4和IL-1、免疫球蛋白G受体识别的免疫复合物和补体等。TAMs的募集主要涉及CCL2-CCR2轴、CXCL12-CXCR4轴和CSF1-CSF1R轴等通路^[7]^。Wu等^[43]^研究发现，丹参中的丹参酮I通过抑制CCL2/STAT3轴阻止肺癌细胞招募巨噬细胞，减少靶器官中TAMs的数量，降低CCL2的分泌，从而发挥抗癌作用。Xu等^[44]^发现中药复方清热活血方可能通过阻断CXCL12/CXCR4/JNK2/STAT3信号通路减少TAMs的募集，降低Arg-1表达水平，升高iNOS水平，抑制VEGF和CD31蛋白的表达，减少肺癌的血管生成。这些中药可以通过减少TAMs被募集至转移靶器官，从源头上减少TAMs的数量，降低转移发生的可能性。

### 4.2 抑制M2型极化

M2型TAMs能促进肿瘤的发生发展，所以调控巨噬细胞极化对于抑制肺癌转移具有重要意义。Xu等^[45]^发现黄芪甲苷IV能够通过抑制巨噬细胞中AMPK信号通路的激活，抑制AMPKα的表达，从而抑制M2型巨噬细胞的极化，减少小鼠体内转移灶数目，降低M2型巨噬细胞浸润瘤体。青蒿素衍生物双氢青蒿素可以通过Akt/哺乳动物雷帕霉素靶蛋白（mammalian target of rapamycin, mTOR）信号通路减少Lewis肺癌细胞（Lewis lung cancer, LLC）小鼠肺脏中TAMs的浸润，显著增加TAMs中M1/M2的比例，此外还能增强M1型巨噬细胞的吞噬能力^[46]^。Zhao等^[47]^发现苦参碱可以抑制RAW264.7细胞中PI3K/Akt/mTOR，抑制巨噬细胞M2型极化，降低IL-4、Arg-1和IL-10的水平；上调LLC细胞中E-钙黏蛋白（E-cadherin）的表达，下调波形蛋白和N-cadherin的表达，抑制LLC细胞的转移以及EMT。Wang等^[48]^证明玉屏风散通过促进STAT1的磷酸化诱导M1型巨噬细胞极化，激活CD4^+^ T细胞并增强其细胞毒性，抑制原位LLC生长。高莉萍等^[49]^发现经中药复方活血化湿汤处理巨噬细胞后，IL-1β水平升高、CCL18浓度降低，提示活血化湿汤可以抑制巨噬细胞M2型极化。刘怡辰等^[50]^研究证明，益气扶正方可能通过调控CCL2/AMPK/mTOR信号通路中蛋白表达，从而抑制巨噬细胞向M2型转化。还有类似的诸多中药都可以通过调节M1/M2的比例，使巨噬细胞不向具有促肿瘤特性的M2型转化，减少肿瘤复发转移的风险^[51,52]^。

### 4.3 调节TME中的细胞因子和蛋白

在TME中TAMs的活化和功能受到一系列相关细胞因子和蛋白的调控，中药可以通过调节这些细胞因子和蛋白的表达水平调控巨噬细胞从而抑制肺癌转移。中药补骨脂的有效成分补骨脂查尔酮可以通过调节肺癌细胞转移相关蛋白E-cadherin、VEGF-C、MMP-3、MMP-9和N-cadherin的分泌，降低肺癌细胞的迁移和侵袭能力^[53]^。生姜中的6-姜辣素具有改善TME免疫抑制状态的作用，其主要通过上调肺癌组织中M1/M2巨噬细胞的比例，增加干扰素-γ（interferon-γ, IFN-γ）和IL-12的表达，并降低IL-10和TGF-β1的表达水平^[54]^。人参皂苷可以降低小鼠肺癌组织中M2巨噬细胞标志物CD206和VEGF-C的表达水平，并且有可能在TME中将M2型TAMs转化为M1表型，从而降低MMP和VEGF的水平并阻止非小细胞肺癌（non-small cell lung cancer, NSCLC）细胞转移^[55]^。重楼有效成分重楼皂苷VII能激活干扰素基因刺激蛋白（stimulator of interferon genes, STING）/TANK结合激酶1（TANK-binding kinase 1, TBK1）/磷酸化干扰素调节因子3（interferon regulatory factor 3, IRF3）信号通路，促进M1型巨噬细胞分泌促炎细胞因子，从而抑制由IL-10驱动的肿瘤细胞中STAT3的激活及TGF-β的分泌，以旁分泌的方式抑制肿瘤细胞的增殖和侵袭^[56]^。中医经典方剂补肺汤可以通过抑制IL-10和PD-L1的表达，减弱TAMs诱导的NSCLC增殖、迁移、侵袭和免疫抑制^[57]^。通过影响细胞因子和蛋白的表达水平，中药可以多靶点、多途径地干预肺癌转移过程，抑制肿瘤发生发展（表1）。

**表1:** 中药复方及单体调控TAMs抑制肺癌转移的基础研究

Chinese medicine compounds/monomers	Method	Mechanism	Ref.
Dihydroisotanshinone I	Reduced recruitment of TAMs	Inhibited the CCL2/STAT3 axis, reduced the secretion of CCL2	^[43]^
Qing-Re-Huo-Xue formulae	Reduced recruitment of TAMs	Blocked the CXCL12/CXCR4/JNK2/STAT3 signaling pathway	^[44]^
Astragaloside IV	Inhibited M2 polarization	Inhibited activation of the AMPK signaling pathway resulted in suppressed expression of AMPKα	^[45]^
Dihydroartemisinin	Enhanced M1 polarization，Inhibited M2 polarization	Enhanced M1 phagocytic capacity was achieved through augmentation of the M1/M2 ratio via activation of the Akt/mTOR signaling pathway	^[46]^
Matrine	Inhibited M2 polarization	Inhibited PI3K/Akt/mTOR, reduced levels of IL-4, Arg-1 and IL-10; upregulated the expression of E-cadherin, and downregulated the expression of Vimentin and N-cadherin	^[47]^
Yu-Ping-Feng decoction	Enhanced M1 polarization	Enhanced the phosphorylation level of STAT1	^[48]^
Huoxue Huashi decoction	Inhibited M2 polarization	Raised IL-1β concentration while decreased CCL18 concentration	^[49]^
Yiqi Fuzheng prescription	Inhibited M2 polarization	Regulated the protein expression in the CCL2/AMPK/mTOR signaling pathway	^[50]^
Bavachalcone	Regulated cytokines and proteins in the TME	Regulated the secretion of E-cadherin, VEGF-C, MMP-3, MMP-9 and N-cadherin	^[53]^
6-Gingerol	Regulated cytokines and proteins in the TME	Increased the expressions of IFN-γ and IL-12, reduced the levels of IL-10 and TGF-β1	^[54]^
Ginsenoside Rh2	Regulated cytokines and proteins in the TME	Reduced the expression levels of CD206 and VEGF-C; reduced the levels of MMP and VEGF	^[55]^
Polyphyllin VII	Regulated cytokines and proteins in the TME	Activated the STING/TBK1/IRF3 signaling pathway, promoted the secretion of pro-inflammatory cytokines, inhibited the activation of STAT3, and suppressed the secretion of TGF-β	^[56]^
Bu-Fei decoction	Regulated cytokines and proteins in the TME	Reduced the expression of IL-10 and PD-L1	^[57]^

TAMs: tumor-associated macrophages; TME: tumor microenvironment; CCL2: chemokine C-C motif ligand 2; STAT3: signal transducer and activator of transcription 3; mTOR: mammalian target of rapamycin; PI3K: phosphoinositide 3-kinase; IL-4: interleukin 4; Arg-1: arginase-1; VEGF-C: vascular endothelial growth factor C; MMP: matrix metalloproteinase; IFN-γ: interferon-γ; TGF-β: transforming growth factor β; STING: stimulator of interferon genes; TBK1: TANK-binding kinase 1; IRF3: interferon regulatory factor 3; PD-L1: programmed cell death ligand 1.

## 5 总结与展望

现代肿瘤学以肿瘤细胞为中心，围绕肿瘤细胞从原发灶脱落进入外周血成为循环肿瘤细胞，发生血管外渗进入靶器官定植成为播散肿瘤细胞的一系列过程制定诊疗方案，实施精准治疗。近年来免疫疗法的兴起，使得肺癌的治疗思路逐步从“以瘤为主”向“人瘤并重”再到“以人为本”发生转变。免疫功能失调在肿瘤发生发展过程中的重要作用已经得到普遍认可，而很早就有中医学者提出免疫功能与中医“正气”的概念具有高度一致性^[58]^。在中医肿瘤学中，以免疫紊乱为基础的“正虚”致癌已经成为共识。国医大师刘嘉湘先生认为恶性肿瘤的形成主要是由于机体正气不足不能抗邪，气滞、血瘀、痰湿、毒聚等外邪内毒淤积于体内，久之形成局部肿块，实际是一种全身性疾病的局部表现^[59]^。田建辉教授在传承刘嘉湘先生“扶正治癌”学术思想的基础上，创新性地提出了肺癌转移的“正虚伏毒”学说^[60]^，“正虚”主要指免疫衰老、免疫监视功能下降、免疫逃逸等免疫功能紊乱；“伏毒”指潜伏于体内的癌细胞（例如循环肿瘤细胞、DTCs、休眠肿瘤细胞等），具有“正盛则伏而不作，正虚则出而为病”的特征，藏于血道脏腑，流窜全身，伺机为病。故而按照中医学“扶正祛邪”的辩证思路治疗肺癌转移具有科学性和理论支撑。

巨噬细胞的双向调节作用与中医阴阳理论中阴阳的对立制约、互根互用、相互转化和消长平衡等特点并行不悖。在肺癌初期，人体正气尚存，正气可以自主抗邪，这时TAMs中具有抗肿瘤作用的M1型巨噬细胞占主导地位，通过释放多种炎性介质促进肿瘤细胞的清除，与中医理论中“阴主杀”的特性相符合；肺癌进展到中晚期，邪盛正虚，正气无力抗邪，M1型TAMs逐渐向M2型转化，促进肿瘤进展，与中医理论中“阳主生”的特性相一致，这时需使用扶正祛邪的中药来调动和激活免疫系统，调节免疫功能，改善免疫抑制，与中医学的“扶正治癌”原则不谋而合。因此，可以基于中医阴阳理论通过平衡M1与M2的消长关系，控制肺癌的进程。

随着现代医学技术的发展，传统中医药对于靶向巨噬细胞防治肺癌的研究也在逐渐深入。中医药对巨噬细胞的调控方式多种多样，一些中药可以直接减少TAMs的募集、抑制巨噬细胞M2型极化，另一些可以通过调节TME中的细胞因子和蛋白间接地抑制肿瘤的生长、侵袭和转移；特别是在免疫抑制的TME中，中医药依然可以通过调节免疫系统，激活抗肿瘤表型M1，抑制促肿瘤表型M2，改善免疫抑制，重塑免疫平衡，发挥其独特优势（图1）。具有调节巨噬细胞作用的常见中药成分有苷类、生物碱类和多糖类等，它们可以通过一种或多种受体激活MAPKs、核因子κB（nuclear factor kappa-B, NF-κB）、STAT、STING等信号通路，或调控相关细胞因子和蛋白进行肿瘤免疫调节^[61]^。

**图1:**
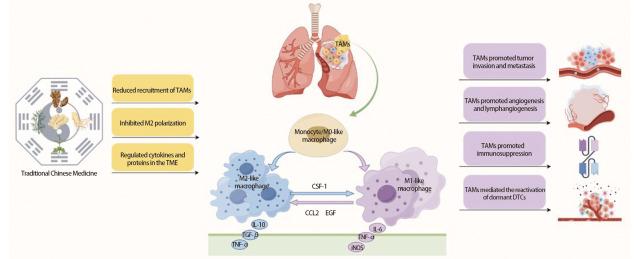
中医药调控TAMs防治肺癌转移的机制图

虽然中医药在靶向巨噬细胞抗肿瘤免疫治疗方面已经取得一些进展，但仍存在许多问题尚未解决，例如许多中药复方调控巨噬细胞的通路较为复杂难以研究、靶向巨噬细胞治疗的临床验证较少且缺乏远期疗效证据、巨噬细胞的极化与主要临床指标肿瘤分期之间是否具有相关性尚不明确等。肺部复杂的微环境支持原发性肺癌和肺外肿瘤转移的发生，但也为治疗提供了许多尚未开发的靶点资源。为了最大程度地提升患者的治疗效果，可以将西医的放化疗、靶向治疗、免疫治疗与传统的中医药治疗方法相结合，联合疗法将是未来重要的研发方向。中医药从整体观念入手，具有多靶点、多机制的治疗优势，也将在未来进行更多的深入研究和探索，以期突破肿瘤转移的瓶颈。
